# Moving toward Soft Robotics: A Decade Review of the Design of Hand Exoskeletons

**DOI:** 10.3390/biomimetics3030017

**Published:** 2018-07-18

**Authors:** Talha Shahid, Darwin Gouwanda, Surya G. Nurzaman, Alpha A. Gopalai

**Affiliations:** School of Engineering, Monash University Malaysia, Jalan Lagoon Selatan, Bandar Sunway 47500, Malaysia; talha.shahid@monash.edu (T.S.); surya.nurzaman@monash.edu (S.G.N.); alpha.agape@monash.edu (A.A.G.)

**Keywords:** hand exoskeletons, soft robotics, rehabilitation, activities of daily living, systematic and chronological review

## Abstract

Soft robotics is a branch of robotics that deals with mechatronics and electromechanical systems primarily made of soft materials. This paper presents a summary of a chronicle study of various soft robotic hand exoskeletons, with different electroencephalography (EEG)- and electromyography (EMG)-based instrumentations and controls, for rehabilitation and assistance in activities of daily living. A total of 45 soft robotic hand exoskeletons are reviewed. The study follows two methodological frameworks: a systematic review and a chronological review of the exoskeletons. The first approach summarizes the designs of different soft robotic hand exoskeletons based on their mechanical, electrical and functional attributes, including the degree of freedom, number of fingers, force transmission, actuation mode and control strategy. The second approach discusses the technological trend of soft robotic hand exoskeletons in the past decade. The timeline analysis demonstrates the transformation of the exoskeletons from rigid ferrous materials to soft elastomeric materials. It uncovers recent research, development and integration of their mechanical and electrical components. It also approximates the future of the soft robotic hand exoskeletons and some of their crucial design attributes.

## 1. Introduction

The emerging trend of soft robotics has stimulated the interest of engineers and researchers around the world to look into various applications, ranging from biomedical and rehabilitation to grasping and manipulation [1,2]. Biomimetic and bioinspired soft robots have been among the most successful products of soft material robotics. Among others, the inspiration for these soft robots originates from examining invertebrates like caterpillars, worms and fish grubs [2]. The hydrostatic and fluid-like structure motivates researchers to look more into the use of soft materials to develop similar structures. 

One of the major lessons learned from these biostructures was the ability to form and adapt to complexly shaped bodies. This led to various developments such as: (1) an octopus-like robot for flexible manipulation [2]; (2) a worm-like robot that uses a thermal shape-memory alloy (SMA) actuator to imitate the motion of its biological counterpart [3]; and (3) a caterpillar-shaped soft robot that imitates the process of translating deformation to locomotor dynamics [4].

Another important development in the emerging field of soft robotics is the use of pneumatic soft grippers for handling fragile objects such as an uncooked egg or an anesthetized mouse [5]. These devices can grip, hold and release certain complexly shaped objects. They have several fingers to hold delicate objects by intelligently adapting themselves to the shape of the object and providing maximum gripping force without damaging it.

This new trend is especially interesting for biomedical and rehabilitation engineering applications as well, with the hand exoskeleton as one of the examples. A major shift from the use of hard to soft materials can be observed in some of the latest designs of hand exoskeletons, such as the Wyss Institute glove [6,7,8], the Magnetic Resonance Compatible (MRC) glove [9,10,11,12], the National University of Singapore (NUS) glove [13,14,15,16,17] and the Seoul National University (SNU) glove [18,19,20,21,22,23]. The hand exoskeleton is an integral part of rehabilitation robotics that provides rehabilitation exercises and assistance in activities of daily living (ADL), such as gripping and grasping [24]. It is commonly recommended for patients with cerebrovascular disease [24], cerebral palsy [25] and rheumatoid arthritis [26].

**Figure 1:**
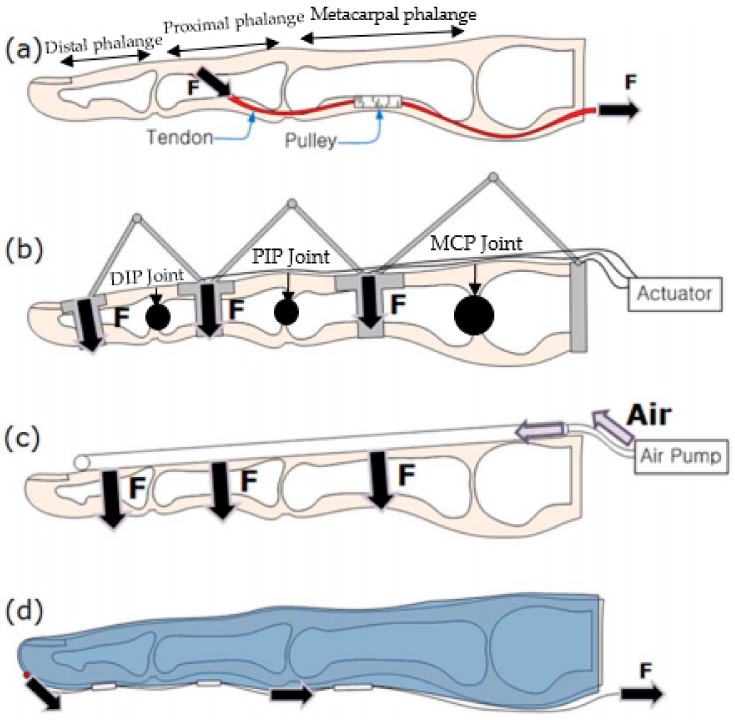
Internal bone structure of a human finger and finger exoskeleton robot, where ‘F’ is the actuation force applied in its respective direction (arrow head). (**a**) The natural skeletal structure of the human finger. (**b**) The rigid joint and link mechanism of a hand exoskeleton; distal (DIP), proximal (PIP) and metacarpal (MCP) interphalangeal joints. (**c**) The pneumatically-actuated hand exoskeleton. (**d**) The tendon/cable–pulley system in a wearable glove. Reprinted and modified with permission from [27], published under the Creative Commons Attribution license (CC BY 2.0) [32].

Unlike a prosthetic hand, a hand exoskeleton is designed and built around the human hand; thus, it has to conform to the hand anatomy and its range of motion to minimize the wearer’s discomfort. More importantly, it has to be light and able to be put on easily so that the wearer can use it daily to perform basic activities. Figure 1a shows the natural skeletal structure of the human finger, the movement of which may be assisted. The structure consists of three joints: distal (DIP), proximal (PIP) and metacarpal (MCP) interphalangeal joints [27,28]. The finger movements are controlled through the activation of extrinsic and intrinsic muscles. The extrinsic muscles are actuated from the forearm and control the flexor and extensor muscle tendons to move the fingers. The intrinsic muscles are located within the finger, and they control the independent motion of the finger [28]. The maximum flexion for the MCP joint ranges from 70° to 95° depending on the finger orientation, while the maximum flexion for the DIP and PIP joints is about 110° and 90°, respectively.

Hand exoskeletons have endured extensive research, primarily in the field of assistive and rehabilitative robotics, and have also been discussed from various perspectives [29,30,31]. Several iterations of different hand exoskeletons indicate the growing need for a better, lighter and more practical solution. Most of the existing hand exoskeletons adopt one of the design approaches depicted in Figure 1. With the rise of soft robotics, there has been a progressive shift from the conventional rigid mechanical structure designs (Figure 1b) to designs with softer actuation (Figure 1c) and designs that closely resemble the natural finger musculoskeletal structure (Figure 1d) [27]. This study aims to review these changes in the recent decade and discuss how the adoption of soft robotics helps in designing a more compliant hand exoskeleton. The design of a hand exoskeleton can be divided into three main components: the mechanical design, the actuation unit and sensory feedback control. This work also examines how soft robotics technology has changed the architectures of these components over the years.

## 2. Method

A chronological review was conducted of several renowned literature databases, i.e., Science Direct, PubMed, IEEE Xplorer, SciVal and Google Scholar, to identify the trend of soft robotics in hand exoskeletons from the year 2008 onward. The keywords used as the search entries to examine more categorical papers were ‘soft hand exoskeleton’ and ‘soft robotic hand exoskeleton’. The search results were further filtered with keywords like ‘rehabilitation’, ‘ADL’, ‘pneumatic’ and ‘tendon’. A set of inclusion and exclusion criteria was then applied to select the right literature.

### 2.1. Inclusion and Exclusion Criteria

The following inclusion and exclusion criteria were used to funnel down the discrete knowledge that is vital for the systematic review and chronological review of the soft robotic hand exoskeleton.

#### 2.1.1. Inclusion Criteria

- The study introduced and applied soft robotics technology for the development of a hand exoskeleton that included a wearable glove, actuator or both
- The study presented the mechanical or electrical aspect of the hand exoskeleton in at least three of the following attributes: actuation, active degrees of freedom (DOFs), finger movement, output force, range of motion (ROM), weight and functionality
- The hand exoskeleton had at least one soft robotic finger (3 degrees of freedom)
- The study presented a prototype or at least a design of the hand exoskeleton

#### 2.1.2. Exclusion Criteria

- The study was published in any language other than English
- The device used rigid or ferrous mechanical components for the glove design and linkages
- The device was used other than that for rehabilitation and assistance for ADL
- The device was a prosthetic or anthropomorphic hand

A total of 65 literature works on soft robotic hand exoskeleton were found. However, only 45 devices were selected for the systematic review, because some designs were reported in several articles and used for different purposes. For example, the SNU research group presented their Exo-Glove Poly in [20,21,22] and Exo-Poly PM, which has a similar design approach as in [33]. Another example is the NUS research group, who originally developed the MRC glove for functional magnetic resonance imaging (fMRI) in 2015 [11] and reported its use for stroke patients in 2016 [12]. It is important to note that hand exoskeletons, which have rigid actuation or rigid wearable linkages, were excluded from this study. Prosthetic and anthropomorphic hands were excluded because they have a gripper that can be replaced or mounted on a stump or wearable object. Last but not least, even though the function of prosthetic and anthropomorphic hands are quite similar to that of a hand exoskeleton, this study focused on exoskeletons designed mainly for rehabilitating and assisting individuals with medical conditions such as stroke and muscular deformity.

### 2.2. Methodological Framework

Previous reviews revolved around short-term narration [29] and general challenges [30] of the soft robotic hand exoskeletons. As the number of literature works on this system has grown over the recent decade, it is important to review how they adopt soft robotic technology and evolve from the use of hard materials and rigid linkages to soft materials and actuation, to overcome the limitations posed by conventional hand exoskeleton designs. The framework for this research was taken forth into two areas: a systematic review of the soft robotic hand exoskeleton features and a chronological analysis to identify the emerging trends in hand exoskeletons.

The systematic review describes the merits and demerits of different exoskeletons based on their design attributes, such as ergonomics, complexity, instrumentation and actuation mode. It also discusses different design techniques that distinguish one soft exoskeleton from another. For this purpose, the attributes selected for comparison between different exoskeletons were based on quantitative results such as year of publication, mode of actuation, number of fingers, active degrees of freedom, output force and many other attributes.

The chronological review investigates the adoption of soft robotics technology in the design of hand exoskeletons in the recent decade. Here, we review the evolution of the soft robotic hand exoskeleton systems and how the newer designs use soft robotics as a form of improvement from the previous studies. We examine the exoskeleton mechanical and electrical attributes and their functionalities based on the articles published from 2008 to 2017. It is hoped that by evaluating these elements annually, one can realize the importance of soft robotics in designing and developing better hand exoskeletons.

## 3. Soft Hand Exoskeleton Systems

Forty-five soft robotic hand exoskeletons were systematically reviewed based on the criteria described in Section 2. Hand exoskeletons from the same research group with similar attributes and yet different years of publication were merged into one group. They were organized in chronological order, then by their mode of actuation and output force (Table 1). This section is further divided into two parts. The first part discusses the general trend in soft robotic hand exoskeletons, or soft wearable gloves, while the second part discusses the actuation mechanism in more detail.

### 3.1. The General Trend: Soft Wearable Glove

Unlike prosthetics, hand exoskeletons are designed to actuate or mechanize the paralyzed human hand. In its early years, hand exoskeletons were made of rigid linkages. These linkages connected the exoskeleton with the finger, and the rotational axes of the joints were aligned with the human finger joints (Figure 1b). Despite its wider adoption in clinical and rehabilitation settings, this design is heavy, and misalignments between the human finger and robot rotational axes are common [27]. Therefore, in recent years we have seen the use of “soft robotic hand exoskeletons” (or sometimes referred to as “soft wearable gloves”) as a general trend and as one of the plausible solutions that would overcome the limitations posed by a hard material exoskeleton. A soft wearable glove can be defined as a wearable glove made of soft materials and possessing a particular mechanism such that it can be actuated like a natural hand. One of the advantages of a soft wearable glove is that it can be made light and compact so that it can be worn and used comfortably. However, it is also important to ensure that it can transmit the intended forces to the fingers. Generally, it is argued that soft wearable gloves can reduce the weight of the hand exoskeleton system and allow a compact self-alignment of robot joints with the finger joints’ rotational axes [20].

**Figure 2:**
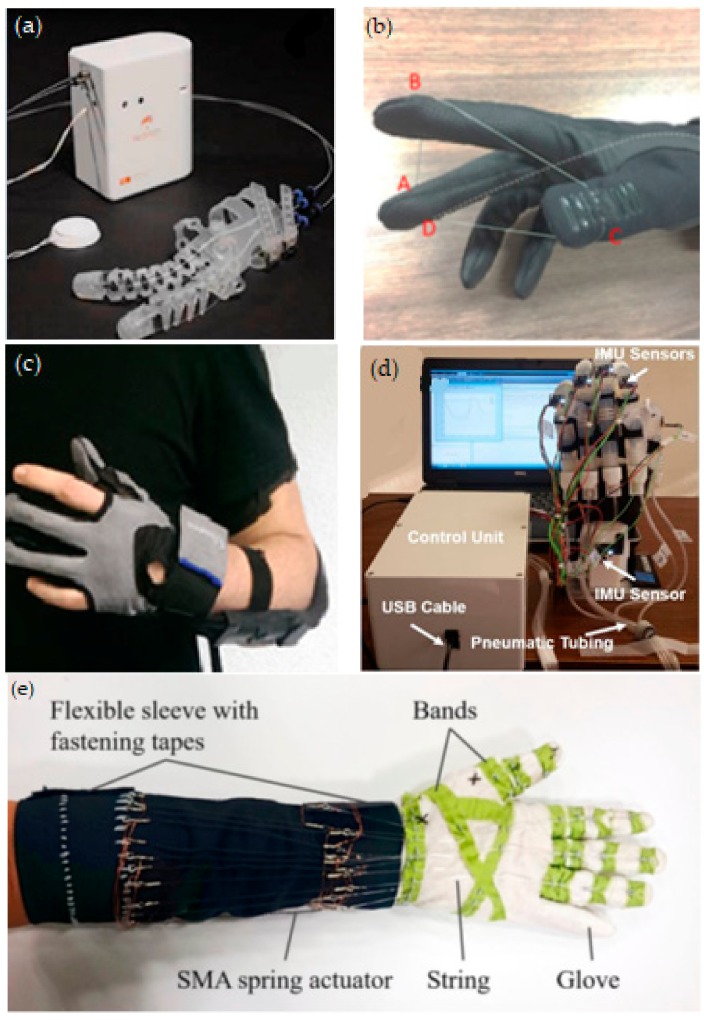
Soft wearable gloves for rehabilitation and ADL. (**a**) Exo-Glove Poly 2.0 (improved version). Reprinted from [52] with permission from Springer Nature. (**b**) Three fingered GRIPIT with a single actuator with cable routing across the point ‘A to D’ indicated in red. Reprinted with permission from [23] published under the Creative Commons Attribution license (CC BY 4.0) [78]. (**c**) The iHand system. Reprinted from [59] with permission from Springer Nature. (**d**) Jarayani glove bending with control unit, Inertial Movement Unit (IMU) sensor and Universal Serial Bus (USB) cable. Reprinted from [48] with permission from IEEE. (**e**) Prototype of the muscle glove using shape-memory alloy (SMA) spring actuators. Reprinted from [74] with permission from Springer Nature.

Soft wearable gloves are usually made with fabric, plastic or silicone-based material. They can be designed as per the patient’s finger measurements. The fabric-based materials can be seen in the earlier designs of the SNU Exo-Glove [10], the glove by Nilsson et al. [40] and the RoboGlove [51]. However, the linkages need to be stitched to the fabric, which is vital for nullifying the slippage of the glove during flexion and extension. Another limitation of this design is that during flexion, the glove can roll up around the PIP joint because the fabric material is less elastic than human skin. This creates the need for a more elastic, but durable soft material that can replace the fabric of the soft wearable glove. This was mentioned in [18] and was realized in its successive iteration: the Exo-Glove Poly 2.0 (Figure 2a) [52]. In this new design, the rigid linkages and the wearable glove are made of elastomers, while the cables are routed in polytetrafluoroethylene (PTFE) tubing.

A tendon/cable-driven mechanism is one of the most common actuation mechanisms embedded in the soft glove and generally requires more than one actuator. In the earlier designs, this mechanism had actuator units for each flexion and extension of the finger [22]. The tendons/cables pass across the fingers via cable routing, which connect the motor with the tip of the fingers. One of the major advantages in this approach is routing, which allows for multiple motions with a single actuation [18,19]. To overcome some of the design challenges, the soft glove generally needs to be constrained with a thimble [18]. The thimble is a part of the glove that encompasses the distal phalange firmly and acts as an anchor point for the tendon/cable. As the motor rotates, the tendon then forces the thimble to move in the intended direction and subsequently flexes or extends the finger. Other than the thimble, this design needs a tendon anchor support as well, and can be found in the Exo-Glove Poly 2.0 [18], the SNU glove [20] and the Robonaut 2 (R2) glove [51]. This support anchors the string to a fixed rigid body, usually a three-dimensional (3D)-printed part that is attached on the dorsal side of the palm. Another innovative soft glove design, which is worth noting here, is the GRIPIT glove (Figure 2b) [23]. This design involves wires routed around the fingers and capstan. The wires are then connected to the spool of the motor to control the tension and allow the finger to flex in a perfect pinching position. It is not necessary to use a tendon/cable–pulley system to imitate the natural motion of a finger. Yao et al. [74] used SMA springs to actuate the finger into flexion or extension and to retain its resting position. The iH glove (Figure 2c) uses a cable-actuated wearable glove to support patients in performing ADL [59].

Another common actuation mechanism is pneumatic based, in which most of the soft pneumatic actuators have strain limiting layers and/or fibers. The NUS glove [16], the MRC glove [11] and the Wyss Institute glove [7] inserted these layers into their actuators to allow the finger to flex appropriately, while, on the other hand, Exo-Glove PM [33] constrains the expansion of the air chamber in a specific direction, thus enabling the finger to flex during actuation. Haghshenas-Jaryani et al. [48] proposed an actuator that has ridge-like shapes in it (Figure 2d). These ridges can expand due to air pressure and force the finger to curl. This design is reported to have a larger bending angle and faster stroke at a pressure lower than other actuators. However, it only has mediocre gripping force. Another innovative biomimetic design can be seen in the soft muscle glove (Figure 2e). This device uses SMA springs and tendon actuators to control the motion of the wearable glove, and its motion is similar to natural hand motion [74].

### 3.2. Discussion of the Actuating Mechanism

**Figure 3:**
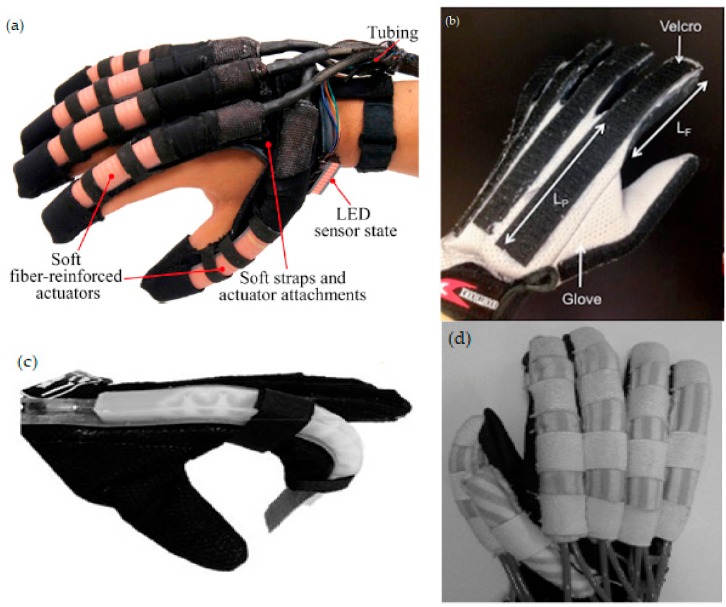
Different actuation mechanisms for soft robotic hand exoskeletons. (**a**) The Harvard Glove 2.0 with fiber-reinforced actuators with fiber-reinforced actuators and light-emitting diode (LED) sensor display. Reprinted from [6] with permission from Elsevier. (**b**) The NUS prototype glove with fabric as the strain limiting layer. Reprinted from [10] with permission from Taylor & Francis. (**c**) The NUS glove for rehabilitation. Reprinted from [17] with permission from Springer Nature. (**d**) The Power-Assist glove. Reprinted from [38] with permission from Fuji Technology Press Ltd.

An actuator is generally defined as having the ability to convert energy into motion. Actuators in a soft robotic hand exoskeleton mainly are comprised of tendon/cable–pulley and pneumatic/hydraulic systems. These actuators are usually placed on the dorsal side or on the sagittal plane of the finger to maximize the power transmission to the fingertip. In the pneumatic system, the air chamber is placed during the fabrication process. The soft actuator is then placed on the dorsal side of the fingers by either attaching it to the hand through a harness (e.g., Velcro straps), stitching it to fabric-based material or gluing it to a glove [6]. The most popular pneumatic soft robotic hand exoskeletons are the Wyss Institute glove [8] and the MRC glove [11]. Both designs embed the pneumatic/hydraulic chamber within the fabrication process. The soft material is fabricated in a multi-layer process and has different strain limiting layers and/or fibers as constraints to deliver the required torque during actuation (Figure 3a,b). These layers are usually made of paper or glass placed on top of the soft material, to induce translational constraints, while strain fibers are coiled around the soft material in a clockwise and anticlockwise fashion to limit its rotational motion at its axis [8]. These designs are commonly used for continuous passive motion and active rehabilitation exercises because they are heavy and bulky [30]. However, a pneumatic actuator offers several advantages over the tendon/cable-driven mechanism. One of them is its control strategy [11]. Since the pneumatic pressure is uniformly applied on the entire region, it is relatively easy to measure, thus control, the kinematics of the device.

Generally, the control strategies of the hand exoskeletons can be divided into two categories: low-level and high-level control strategies. In the low-level control strategy, the controller addresses the physical parameters of the device such as force, torque and position [79]. It uses basic control loops for positioning or controlling force/torque based on the design parameters. The high-level control strategy is sometimes referred to as the impedance or admittance control strategy in which it measures the exoskeleton and the environment. A high-level controller can handle a low-level controller in performing the positioning- and force-related tasks [79]. It can use information from low-level controllers to evaluate various factors like gripping force, finger positioning and bending angle, simultaneously. It also allows better estimation of values; for example, the shape of the object, the required gripping force needed to hold the object, the stroke speed and many other factors [77]. This advantage facilitates the control of position as per the shape of the object and applies the required forces to a near perfect grip [79].

Another important aspect in designing a hand exoskeleton is the weight, which is also related to the reason behind choosing the actuating mechanism. As can be seen in Table 1, most of the soft robotic hand exoskeletons weigh below 500 g, which is an allowable weight for ADL [18]. However, the problem lies with the actuator unit, which includes the compressor, which may weigh more than 3 kg [46]. Therefore, pneumatic actuators are less favorable for ADL since it is necessary to carry the actuator unit in a backpack [18]. However, it can be used for rehabilitation purposes. This can be seen in the NUS glove in Figure 3c, which was designed for stroke patients. This glove has single-channel actuators for finger rotation and dual-channel actuators on the wrist to enable better active resistive rehabilitation therapy [45].

Most of the hand exoskeletons designed for clinical research purposes are pneumatically-actuated [59], such as the MRC glove [9,11], Rheumatoid Arthritis Rehabilitative Device (RARD) [70], Rheumatoid Arthritis (RA) glove [26] and the glove by Reymundo et al. [65]. The MRC glove was designed to measure the neurological development in a physiotherapy session [11]. A soft robotic hand exoskeleton was advantageous to use because of the absence of metal interference during neuroimaging [11]. The RARD and RA gloves were designed specifically for patients suffering from rheumatoid arthritis [26,70]. The uniform force distribution on different phalanges allows soft pneumatic actuators to be used in various task-specific training programs during therapy.

One of the limitations of a soft pneumatic actuator is the delivered force or torque. The embedded pressure chamber requires high-pressure air to inflate and transmit the force to the end effector. While a driving force can be initiated, it is hard to control the holding force (gripping force). As the holding position requires continuous force, the pneumatic actuator has to continuously supply a specific amount of pressure to sustain the required gripping force [33]. The power losses during the holding position are much larger than tendon/cable actuators as well. This makes pneumatic actuators less popular than the other actuators. In the tendon/cable–pulley mechanism, the actuator unit simply needs to maintain the tension in the cable, which can be controlled by the capstan adjustment unit [20]. Table 1 affirms the differences between the output forces of tendon/cable-driven actuators and pneumatic actuators. Although the minimum force required for gripping equals 10–13 N [50], cable-driven actuators require less power for continuous holding force (gripping) than pneumatic actuators.

## 4. Technological Trend

The second part of this review paper is to investigate the evolution of soft robotic hand exoskeletons in the recent decade. It highlights the areas that have been investigated and the contributing factors in opening new avenues for further research. It also evaluates the trend and predicts how soft robotic hand exoskeletons may look in the future. Another important aspect of this review is to explore different parameters and variables used as the design criteria to develop hand exoskeletons.

### 4.1. Timeline of Exoskeleton

Sixty-five articles were carefully reviewed and are listed chronologically in Table 2 based on their attributes. Research groups like SNU [18,19,20,21,22,23], NUS [12,13,14,15,16,17], MRC [9,10,11] and The Wyss Institute [6,7,8] are among some of the renowned groups that have published several conference proceedings and journals based on similar principles and design concepts, yet exhibiting new approaches and results throughout the last decade.

One of the earliest works on soft robotic hand exoskeletons was the Power-Assist glove reported in [34] in 2008. It has electromyography (EMG) and low-level controller to actuate a five-fingered glove. The purpose of this glove was to assist patients in ADL. It uses a fabric-based material as the outer shell to encompass the pneumatic actuators. This material also acts as a constraint to force the actuator to bend and assist hand motion [34]. In 2009, the J-glove was introduced and used by post-stroke patients for therapeutic purposes [35]. In 2010, an approach that took advantage of virtual reality for rehabilitation of stroke patients was described in [37]. The PneuGlove [37] has a high-level control strategy including proportional–integral–derivative (PID) control and a virtual reality interface. It aims to reintegrate the eye–hand coordination that is damaged due to stroke or any spinal cord injury.

In 2011, three articles were published. One of them was an exoskeleton that uses the tendon/cable–pulley mechanism [36], while the rest describe the improved Power-Assist glove (Figure 3d) [38,39]. This new glove uses EMG and other sensors to activate the glove and to control different gripping and grasping activities. Experiments were conducted on five patients with different medical conditions, and improvements during their respective therapy sessions were observed.

In 2012, three studies were found, and all of them were tendon/cable-actuated soft exoskeletons [20,21,40]. One of them is the SNU Exo-Glove Poly 1.0 [20,21]. Kinematics and kinetics analysis of the glove in transmitting the force during finger flexion and extension were investigated [20,21]. The other study is the Soft Extra Muscle (SEM) glove by Nilsson et al. [40]. This glove is comprised of a three-fingered cable-driven hand exoskeleton and a high-level feedback controller. It is used for task-specific training and rehabilitation.

A five-finger cable-driven hand exoskeleton was proposed in 2013 [41]. It has a high-level controller that uses surface EMG to flex and extend the finger. The first Exo-Glove Poly was introduced in the same year and was considered to be the first joint-less wearable device (JLWD) [22]. The soft gripper was also introduced in [8] and was later developed to be the Harvard Glove 1.0. However, neither these designs are included in this study because they do not fit the inclusion and exclusion criteria.

Three pneumatically-actuated exoskeletons were reported in 2014 [43,44,45]. One of them is an extension of the PneuGlove [37] and was published by Coffey et al. [44]. The new PneuGlove can be controlled using EEG. It also provides offline and online training sessions with somatosensory feedback. The second study is an exoskeleton presented by Wang et al. [45]. It uses a polyester mesh sleeve with a crescent shape placed on the dorsal side of the finger. This design allows the air to inflate the chamber and applying pressure and forcing the proximal phalange to a rotary motion. The third study details the use of pressurized fluid instead of air for the exoskeleton [43]. It was designed specifically for thumb rehabilitation.

Since 2015, the development of soft robotic hand exoskeletons escalated. Thirteen studies were published, in which 10 of them were on pneumatic actuators [6,7,11,13,17,19,47,53,54,55] and three studies on the tendon/cable-driven mechanism [19,42,51]. The SNU research group proposed a unique cable routing method for the Exo-Glove. This approach allows the cable to be adjusted to the geometrical properties of the finger [19]. The RoboGlove had its findings published in the same year [51]. The RoboGlove can sustain a load of 680 N and be used in different industries to assist in several delicate tasks. Two articles were published by the Wyss Institute as a continuation of their work on the Harvard Glove [6,7]. This design has a strain limiting layer and strain fibers that enable translation and rotation with a single actuation. It was used for different grasping and gripping activities and was integrated with low- and high-level EMG-based control systems [6,7]. The NUS research group also published their findings on the MRC glove, which was specifically designed for continuous passive motion (CPM) and task-specific training programs [11]. In this design, the strain limiting layer constrained the inflation of air inside the chamber, forcing the finger to rotate in a specific direction.

The research and development of soft robotic hand exoskeletons reached its peak in 2016. Fourteen articles were on pneumatically-actuated exoskeletons [10,12,15,16,48,50,64,65,66,67,68,69,70,71], while eight articles were on tendon/cable-driven systems [18,56,57,58,60,61,62,63]. The SNU research group introduced the Exo-Glove Poly 2.0, which is completely made from elastomer materials [18]. This design has a customized glove that encloses the finger from the thimble at the distal phalange to the metacarpal phalange, thus eliminating the need for fabric-based material as a wearable glove. Park et al. [61] and Xiloyannis et al. [62] published designs for rehabilitation purposes with a focus on the use of a single actuator to control the entire hand. Xiloyannis et al. [63] employed the underactuated control strategy based on the hand postural synergy, while Park et al. [61] introduced exotendon routing to control a pair of fingers simultaneously. Another important development was made by Fischer et al. [63]. This study employed the eXtension Glove (X-Glove) in a clinical study and reported satisfactory rehabilitation results on 15 stroke patients. Yeo et al. [68] integrated strain sensors within the pneumatic actuator during the fabrication process. The strain sensor can be stretched along with the soft actuator to measure forces at different bending angles. The research group from Arlington Research Institute [48,50] adopted a ridge-based design for their five-finger exoskeleton, instead of the use of strain limiting layers and fibers, to achieve translation and rotational motions. This glove [48] showed larger ROM than the Wyss Institute glove [6] with similar output force.

In 2017, eight articles reported the use of pneumatic/hydraulic actuators [9,14,26,33,46,49,76,77], while seven articles presented the use of a tendon/cable-driven system [23,52,59,72,73,74,75]. One of the unique designs uses SMA springs to mimic the function of a finger endoskeleton muscular tendon during flexion and extension motion [74]. The glove weighs only 85.03 g, which is considerably less than many other five-fingered tendon/cable-driven exoskeletons. Chua et al. [26] presented the design of a therapeutic glove for patients suffering from rheumatoid arthritis. In the same year, the SNU group improved the wearability and adaptivity of Exo-Glove Poly 2.0 by introducing a tendon length adjustment module [52]. This allowed the glove to be adjusted to the patients’ hand and finger measurements. The group also published their first pneumatically-controlled wearable glove, Exo-Glove PM [33]. This glove has similar design characteristics as Exo-Glove Poly 2.0 [18]. However, the tendon/cable-driven mechanism was replaced with a pneumatic actuator.

### 4.2. Evolution of the Soft Robotic Hand Exoskeleton

**Figure 4:**
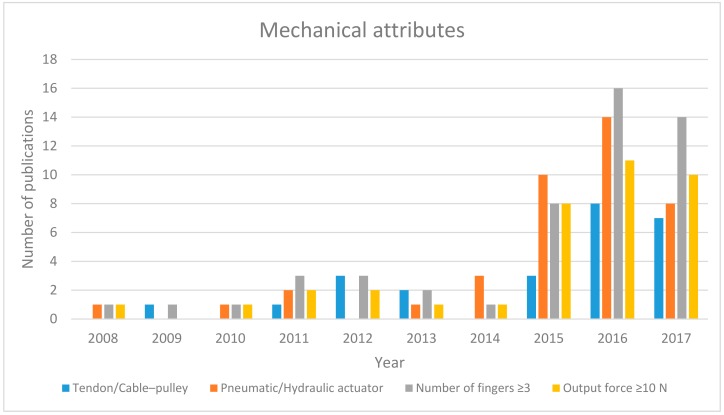
Mechanical development of soft robotic hand exoskeleton devices since 2008.

In order to understand the technological shift in the hand exoskeleton, a chronological analysis was conducted. This analysis is divided into three main areas of interest: mechanical, electrical and functionality of the soft exoskeleton. Figure 4 illustrates the mechanical evolution of soft exoskeletons. The type of actuation, number of fingers and output force are compared. The tendon/cable–pulley actuator dominates the hand exoskeleton design until 2013, (except for the year 2011). The pneumatic actuator was not favored because of its ballooning effect [6]. However, design approaches used in Power-Assist glove [37], PneuGlove [36] and J-glove [39] in 2011 motivated researchers at the Wyss Institute [7,8] and NUS [9,10,11,12,13,14,15,16,17] to focus on overcoming this effect. It produced good results and can be seen in the year 2014, in which all of the research articles reported the use of pneumatic actuator. This trend continued to grow in the next two years. On the other hand, exoskeletons that use tendon/cable–pulley mechanisms show no significant increase in number. Nevertheless, an increase was observed in designs that have equal to or more than three fingers and with output force greater than 10 N. It was also found that not many studies reported the output force of their exoskeletons because studies published in medical journals focused more on the rehabilitation and therapeutic practices rather than the mechanical attributes.

**Figure 5:**
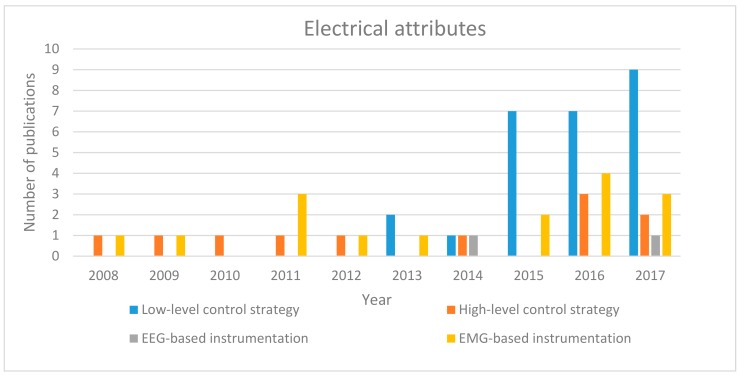
Electric development of soft robotic hand exoskeleton devices since 2008.

The electrical evolution of the soft robotic hand exoskeleton is depicted in Figure 5. The electrical attributes that are of interest here are: high-level and low-level control strategies and the EEG and EMG instrumentations. Similar to the trend observed in Figure 4, the electrical attributes increased from 2015 with more studies reporting the use of low-level controller more than the high-level controller. Furthermore, most of the hand exoskeletons can be controlled by EMG, while only a few of them can be controlled using EEG signals. However, as more studies focus on rehabilitation and therapeutic exercises, reporting on the electrical attributes declined in 2017.

**Figure 6:**
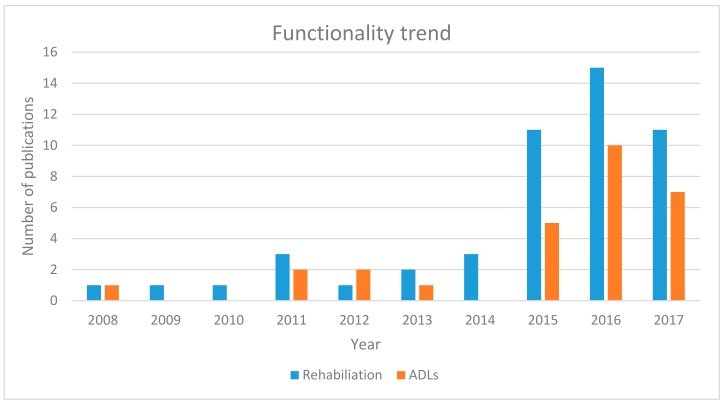
Functionality trend of soft robotic hand exoskeleton devices since 2008.

Figure 6 describes the functionality trend of the exoskeletons since 2008. The trend is devised based on the applications of different soft robotic hand exoskeletons presented in Table 2. Hand exoskeletons are generally developed for two purposes: rehabilitation and assistance in ADL. The functionalities of different soft exoskeletons exhibited a similar trend with mechanical and electrical attributes, especially in 2015 and 2016. However, works published in 2014 focused only on hand exoskeletons for rehabilitation purposes. As the number of designs increased from 2015 to 2017, their functionalities for both rehabilitation and assistance increased at a similar pace.

## 5. Conclusions

Here, we summarize various soft robotic hand exoskeletons based on their merits and demerits and reviews the technological trend of these devices in the last decade, which includes 45 soft wearable exoskeletons along with their design criteria and basic properties. The systematic review describes the general trend and actuation mechanisms of the soft robotic hand exoskeleton. The summary shown in Table 1 presents the mechanical, electrical and functional attributes of different soft robotic hand exoskeletons. The chronological review describes the technological development of exoskeletons and the changes of the design approaches over time. The chronological analysis suggests that the shift of actuation from the tendon/cable–pulley mechanism to pneumatic actuation has increased. Along with that, various research groups have shifted to soft robotics technologies for the design of hand exoskeletons, with an increasing number of research publications since 2015. Based on this review, we conclude that pneumatic-based soft robotic hand exoskeletons have certain advantages compared to the tendon/cable-driven ones, such as their uniform force distribution, while cable-driven actuators also have some advantages, such as less power for continuous holding force. Recent results also showed the potential of a hybrid approach using cable and pneumatic-based actuation [33,49].

Up until now, rehabilitation purposes appear to have led the design and development of soft robotic hand exoskeletons. However, these soft wearable exoskeletons may be used for other functions as well. For example, the virtual reality control used in PneuGlove [37] allows elderly persons improve their brain–limb coordination. This approach can be used to allow other user intent modalities like voice-activated commands to perform certain task-specific training programs. Furthermore, the R2 and RoboGlove were used in manufacturing industries and space exploration programs to perform certain specific tasks [51]. This opens up new avenues of applications for soft robotic hand exoskeletons that are not only specific to rehabilitation and ADL, but also other industrial sectors.

## Figures and Tables

**Table 1:** Summary of the mechanical, electrical and functional attributes of soft robotic hand exoskeletons since 2008.

Year of Publication	Actuation	Output Force (N)/Torque (Nm)	Active Degrees of Freedom	Finger Movement	Range of Motion	Weight	Functionality	Reference
**2008**	Pneumatic	14 N	15	Flexion	-	120 g	ADL	[34]
**2009**	Tendon/cable-pulley	-	15	Flexion	-	-	Task-specific training	[35,36]
**2010**	Pneumatic	2.8 Nm	15	Flexion and extension	-	-	Rehabilitation	[37]
**2011**	Pneumatic	9 N	15	Flexion and extension	90°	120 g	Rehabilitation, ADL	[38,39]
**2012**	Tendon/cable-pulley	20 N	9	Flexion	-	700 g	Task-specific training, ADL	[40]
		18 N	3	Flexion and extension	150°	-	Rehabilitation, ADL	[20,21,22,27]
**2013**	Tendon/cable-pulley	15 N	9	Flexion and extension	-	-	Task-specific training, rehabilitation	[41,42]
	Pneumatic	1.21 N	3	Flexion	-	160 g	Rehabilitation, ADL	[8]
**2014**	Hydraulic	2 N (one finger)	3	Flexion and extension	57.5° (Thumb)	-	Task-specific training	[43]
	Pneumatic	14 N	15	Flexion and extension	90°	-	ADL, rehabilitation, task-specific training	[44]
		4 N	3	Flexion and extension	171°	-	Rehabilitation	[45,46]
**2015**	Hybrid Pneumatic	1.3 N	3	Flexion	150°	-	Rehabilitation, ADL	[47,48,49,50]
	Hydraulic	8 N	15	Flexion and extension	250°	<500 g	Rehabilitation, ADL	[6,7]
	Tendon/cable-pulley	680 N	15	Flexion and extension	-	711 g	Task-specific training, space exploration	[51]
		29.5 N	9	Flexion and extension	112°	194 g	Rehabilitation, ADL	[18,19,52]
	Pneumatic	13 N	3	Flexion	149°	-	Rehabilitation	[53]
		10.35	15	Flexion	141.2°	200 g	Rehabilitation, ADL	[12,13]
		9.25 N	12	Flexion	191.2°	180 g	Rehabilitation, ADL	[9,10,11]
		2 N (one finger)	3	Flexion and extension	143.5°	25 g	Task-specific training,	[54]
		-	3	Flexion	185°	-	ADL	[55]
**2016**	Linear actuator	-	15	Flexion and extension	-	-	Rehabilitation	[56]
	Tendon/cable-pulley	45.42 N (pinch grip)	9	Flexion	-	-	Rehabilitation, ADL	[57,58,59]
		35 N	9	Flexion and extension	-	50 g	ADL	[60]
		32 N	15	Flexion and extension	-	-	Rehabilitation, ADL	[61]
		10 N	8	Flexion and extension	90°	≤500 g	Rehabilitation, ADL	[62]
		-	9	Flexion and extension	93.22°	-	Rehabilitation	[63]
	Pneumatic	35 N	15	Flexion	105.9°	-	Rehabilitation	[64]
		17.7 N	12	Flexion	-	277 g	ADL	[14,15]
		17 N	3	Flexion and extension	93°	-	Rehabilitation	[65]
		17 N	15	Flexion	133°	100 g	Rehabilitation	[66]
		10 N	12	Flexion and extension	-	<100 g	ADL	[67]
		8 N	3	Flexion and extension	-	277 g	Rehabilitation, ADL	[16,17]
		5 N	3	Flexion	-	-	ADL	[68]
		4.66 N	15	Flexion and extension	-	240 g	Rehabilitation	[69]
		2 N	3	Flexion	40°	-	Rehabilitation	[70]
		-	15	Flexion and extension	-	-	Rehabilitation	[71]
**2017**	Tendon/cable-pulley	300 N	5	Flexion and extension	-	800 g	Rehabilitation	[72]
		18 N	9	Flexion	-	40 g	Task-specific training, rehabilitation	[23]
		16 N	15	Flexion	141.2°	300 g	ADL	[73]
		11 N	14	Flexion and extension	96°	85.03 g	Rehabilitation	[74]
		5 N	12	Flexion and extension	110°	285 g	Rehabilitation, ADL	[75]
	Pneumatic	35 N	4	Flexion and extension	90°	-	Rehabilitation	[26]
		22.35 N	9	Flexion and extension	-	350 g	Rehabilitation, ADL	[31]
		4 N	3	Flexion and extension	171°	-	Rehabilitation	[76]
		-	15	Extension	-	-	Rehabilitation	[77]

ADL: Activities of daily living; CPM: Continuous passive motion.

**Table 2:** Number of articles on soft robotic hand exoskeletons since 2008.

Year	Actuators		Number of Fingers ≥ 3	Control Strategy		Instrumentation		Output Force ≥ 10 N	Functionality	
	Tendon	Pneumatic and Hydraulic		Low-Level Control	High-Level Control	EEG	EMG		Rehabilitation, CPM, Task-Specific Training	ADL
**2008**		1 [34]	1 [34]	-	1 [34]	-	1 [34]	1 [34]	1 [34]	1 [34]
**2009**	1 [35]	-	1 [35]	-	1 [35]	-	1 [35]	-	1 [35]	-
**2010**	-	1 [37]	1 [37]	-	1 [37]	-	-	1 [37]	1 [37]	-
**2011**	1 [36]	2 [38,39]	3 [36,38,39]	-	1 [39]	-	3 [36,38,39]	2 [39]	3 [36,38,39]	2 [38,39]
**2012**	3 [20,21,40]		3 [20,21,40]	-	1 [40]	-	-	2 [20,21]	1 [40]	2 [20,21]
**2013**	2 [22,41]	1 [8]	2 [22,41]	2 [8,41]	-	-	1 [41]	1 [41]	2 [8,41]	1 [22]
**2014**		3 [43,44,45]	1 [44]	1 [44]	1 [45]	1 [44]	-	1 [45]	3 [43,44,45]	
**2015**	3 [19,42,51]	10 [6,7,11,13,17,19,47,53,54,55]	8 [6,7,11,13,19,42,51,54]	7 [6,7,11,13,19,51,54]	-	-	2 [7,54]	8 [6,7,11,17,19,42,51,53]	11 [6,7,11,13,17,42,47,51,53,54,55]	5 [6,7,13,19,47]
**2016**	8 [18,56,57,58,60,61,62,63]	14 [10,12,15,16,48,50,64,65,66,67,68,69,70,71]	16 [10,12,15,18,48,56,57,58,60,61,62,63,64,65,67,68]	7 [10,12,15,18,60,64,68]	3 [48,62,65]	-	4 [10,12,60,64]	11 [10,12,15,16,18,60,61,62,64,65,67]	15 [10,12,15,18,48,50,56,57,58,60,61,64,65,68,70]	10 [10,18,50,57,58,61,62,63,64,67]
**2017**	7 [23,52,59,72,73,74,75]	8 [9,14,26,33,46,49,76,77]	14 [9,14,23,26,33,46,49,52,59,72,73,74,75,77]	9 [14,23,26,33,46,73,74,76,77]	2 [9,49]	1 [75]	3 [14,72,74]	10 [9,14,23,26,33,46,72,73,74,75]	11 [9,23,26,33,46,49,72,74,75,76,77]	7 [9,14,33,59,73,75,79]

EEG: Electroencephalography; EMG: Electromyography.
